# Oocyte-Specific Deletion of *Pten* in Mice Reveals a Stage-Specific Function of PTEN/PI3K Signaling in Oocytes in Controlling Follicular Activation

**DOI:** 10.1371/journal.pone.0006186

**Published:** 2009-07-09

**Authors:** Krishna Jagarlamudi, Lian Liu, Deepak Adhikari, Pradeep Reddy, Annika Idahl, Ulrika Ottander, Eva Lundin, Kui Liu

**Affiliations:** 1 Department of Medical Biochemistry and Biophysics, Umeå University, Umeå, Sweden; 2 Clinical Science/Obstetrics and Gynecology, Umeå University, Umeå, Sweden; 3 Medical Biosciences/Pathology, Umeå University, Umeå, Sweden; 4 Department of Chemotherapy, Cancer Center, Qilu Hospital, Shandong University, Jinan, China; 5 Institute of Immunology, School of Medicine, Shandong University, Jinan, China; Harvard Medical School, United States of America

## Abstract

Immature ovarian primordial follicles are essential for maintenance of the reproductive lifespan of female mammals. Recently, it was found that overactivation of the phosphatidylinositol 3-kinase (PI3K) signaling in oocytes of primordial follicles by an oocyte-specific deletion of *Pten (phosphatase and tensin homolog deleted on chromosome ten)*, the gene encoding PI3K negative regulator PTEN, results in premature activation of the entire pool of primordial follicles, indicating that activation of the PI3K pathway in oocytes is important for control of follicular activation. To investigate whether PI3K signaling in oocytes of primary and further developed follicles also plays a role at later stages in follicular development and ovulation, we conditionally deleted the *Pten* gene from oocytes of primary and further developed follicles by using transgenic mice expressing *zona pellucida 3* (*Zp3*) promoter-mediated Cre recombinase. Our results show that *Pten* was efficiently deleted from oocytes of primary and further developed follicles, as indicated by the elevated phosphorylation of the major PI3K downstream component Akt. However, follicular development was not altered and oocyte maturation was also normal, which led to normal fertility with unaltered litter size in the mutant mice. Our data indicate that properly controlled PTEN/PI3K-Akt signaling in oocytes is essential for control of the development of primordial follicles whereas overactivation of PI3K signaling in oocytes does not appear to affect the development of growing follicles. This suggests that there is a stage-specific function of PTEN/PI3K signaling in mouse oocytes that controls follicular activation.

## Introduction

In women, the 300,000–400,000 ovarian primordial follicles at menarche serve as the source of fertilizable ova for the entire duration of reproductive life [1]. In order to ensure the proper length of reproductive life, the majority of primordial follicles remain in a dormant state, and only limited numbers of them are recruited into the growing follicle pool through follicular activation. Menopause occurs when the pool of primordial follicles has become exhausted [1]–[3].

Recently, studies from our research group have suggested that the phosphatidylinositol 3-kinase (PI3K) signaling pathway in oocytes plays an important role in oocyte growth during early follicular development [4]. The PI3K pathway is a fundamental signaling pathway for regulation of cell proliferation, survival, migration, and metabolism [5]. PI3Ks are lipid kinases that phosphorylate the 3′-OH group on the inositol ring of inositol phospholipids. PTEN (phosphatase and tensin homolog deleted on chromosome ten), a lipid phosphatase, dephosphorylates the inositol phospholipids and thus functions as a major negative regulator of PI3K [5].

We have shown that deletion of the *Pten* gene from mouse oocytes of primordial follicles using transgenic mice expressing Cre recombinase mediated by the *growth differentiation factor 9 (Gdf-9)* promoter, results in excessive activation of the entire pool of primordial follicles, which in turn leads to premature depletion of all primordial follicles [6]. However, such mutant mice show normal ovulation and normal litter size in young adulthood before their ovarian follicles are depleted [6].

The current study was designed to investigate the role of PTEN/PI3K–Akt signaling in oocytes of primary and further developed follicles in greater detail. We used a Cre-loxP system to delete the *Pten* gene from oocytes of primary follicles using transgenic mice carrying *zona pellucida 3* (*Zp3*) promoter-mediated Cre recombinase [7]. We found that elevated PI3K–Akt signaling in oocytes of primary and further developed follicles does not affect the pool of primordial follicles. Moreover, female mice lacking *Pten* in oocytes of primary follicles showed unaltered follicular development, ovulation, oocyte maturation, and fertility. Our data indicate that PTEN is probably essential for regulation of follicular activation in a stage-specific fashion.

## Results

### Generation of mice with deletion of *Pten* in oocytes of primary and further developed follicles

**Figure 1:**
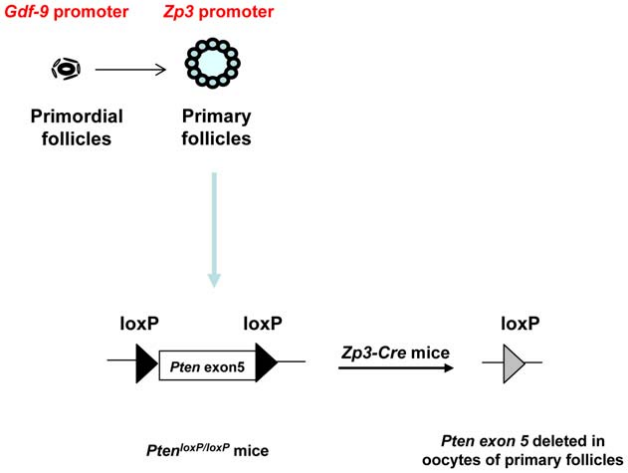
Generation of mutant mice with oocyte-specific deletion of *Pten*. A schematic representation of deletion of *Pten* exon 5 in oocytes of primary and further developed follicles by using the *Zp3* promoter-mediated Cre transgenic mice. The developmental stages at which the *Gdf-9* promoter and the *Zp3* promoter become active are indicated above the illustration of follicles in the figure.

**Figure 2:**
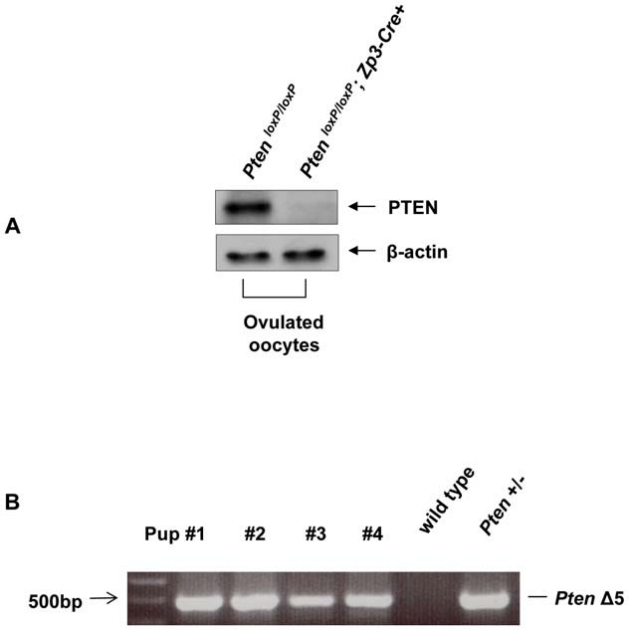
Characterization of *Pten* deletion by western blot and PCR. (A) Oocytes were prepared and lysed for western blot as described in *Materials and Methods*. PTEN expression was found to be completely absent in *Pten^loxP/loxP^*; *Zp3-Cre+* oocytes. For each lane, 150 oocytes were used. β-actin was used as internal control. (B) PCR analysis showing the complete deletion of *Pten* exon 5 (*Pten* Δ5) in one allele of the genomic DNA of pups from *Pten^loxP/loxP^*; *Zp3-Cre+* females.

To inactivate the *Pten* gene from oocytes of primary follicles, we crossed *Pten^loxP/loxP^* mice [8] with transgenic mice carrying *Zp3* promoter-mediated Cre recombinase (*Zp3-Cre* mice) [7], where the *Zp3* promoter drives the expression of Cre recombinase in oocytes from the primary follicle stage (Fig. 1). To determine the efficiency of deletion of *Pten* in oocytes of developing follicles, oocytes were collected by puncturing pregnant mare serum gonadotropin (PMSG)-treated ovaries, followed by western blot analysis. The expression of PTEN protein was found to be completely abolished in *Pten^loxP/loxP^*; *Zp3-Cre+* oocytes (Fig. 2A).

We also confirmed the deletion of *Pten* gene from pups of *Pten^loxP/loxP^*; *Zp3-Cre+* females by PCR. *Pten^loxP/loxP^*; *Zp3-Cre+* female mice were mated with wild-type male mice and their offspring were genotyped for the deletion of *Pten* exon 5 in their tail-tip DNA. As shown in Fig. 2B, the pups showed deletion of *Pten* exon 5 (*Pten* Δ5) in one allele of their genomic DNA. These results indicate that the *Pten* gene was successfully deleted from growing oocytes of *Pten^loxP/loxP^*; *Zp3-Cre+* mice.

### Elevated PI3K–Akt signaling in *Pten^loxP/loxP^*; *Zp3-Cre+* oocytes

**Figure 3:**
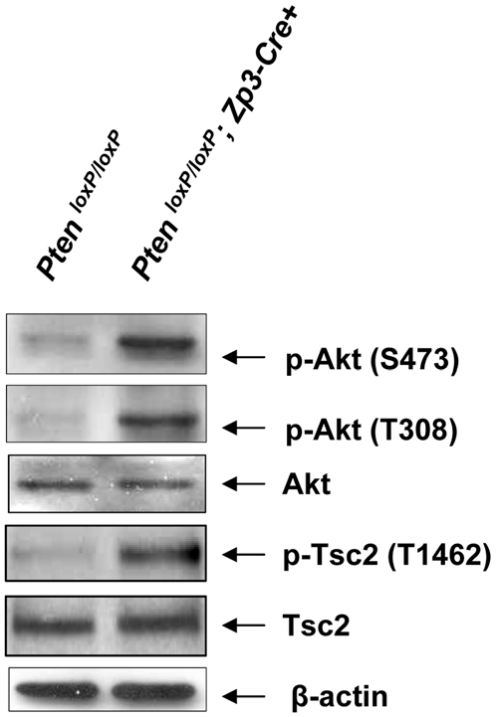
Enhanced Akt signaling in oocytes of *Pten^loxP/loxP^*; *Zp3-Cre+* mice. Oocytes were prepared from ovaries of 3–4 week old mice that were treated with PMSG, as described in *Materials and Methods*. Signaling studies in *Pten^loxP/loxP^*; *Zp3-Cre+* oocytes showed elevated levels of p-Akt (Ser473), p-Akt (Thr308), and p-Tsc2 (Thr1462) as compared to *Pten^loxP/loxP^* oocytes. Levels of total Akt, Tsc2, and β-actin were used as internal controls. 100–150 oocytes were used for each lane. All experiments were repeated at least three times and representative results are shown.

We tested whether the PI3K–Akt signaling pathway is enhanced in *Pten^loxP/loxP^*; *Zp3-Cre+* oocytes. As shown in Fig. 3, phosphorylation of Akt (at Ser473) was found to be elevated in *Pten^loxP/loxP^*; *Zp3-Cre+* oocytes as compared to control oocytes. The phosphorylation of another important site on Akt, Thr308, which is required for complete activation of Akt, was also elevated upon deletion of *Pten* in growing *Pten^loxP/loxP^*; *Zp3-Cre+* oocytes. As an indicator of Akt activity, the phosphorylation of Tsc2 (at Thr1462), which is an Akt substrate, was found to be elevated in *Pten^loxP/loxP^*; *Zp3-Cre+* oocytes compared to control *Pten^loxP/loxP^* oocytes. These results suggest that there is enhanced activation of PI3K–Akt signaling in oocytes of growing follicles upon deletion of *Pten* in *Pten^loxP/loxP^*; *Zp3-Cre+* mice.

### Normal follicular development of *Pten^loxP/loxP^*; *Zp3-Cre+* mice

**Figure 4:**
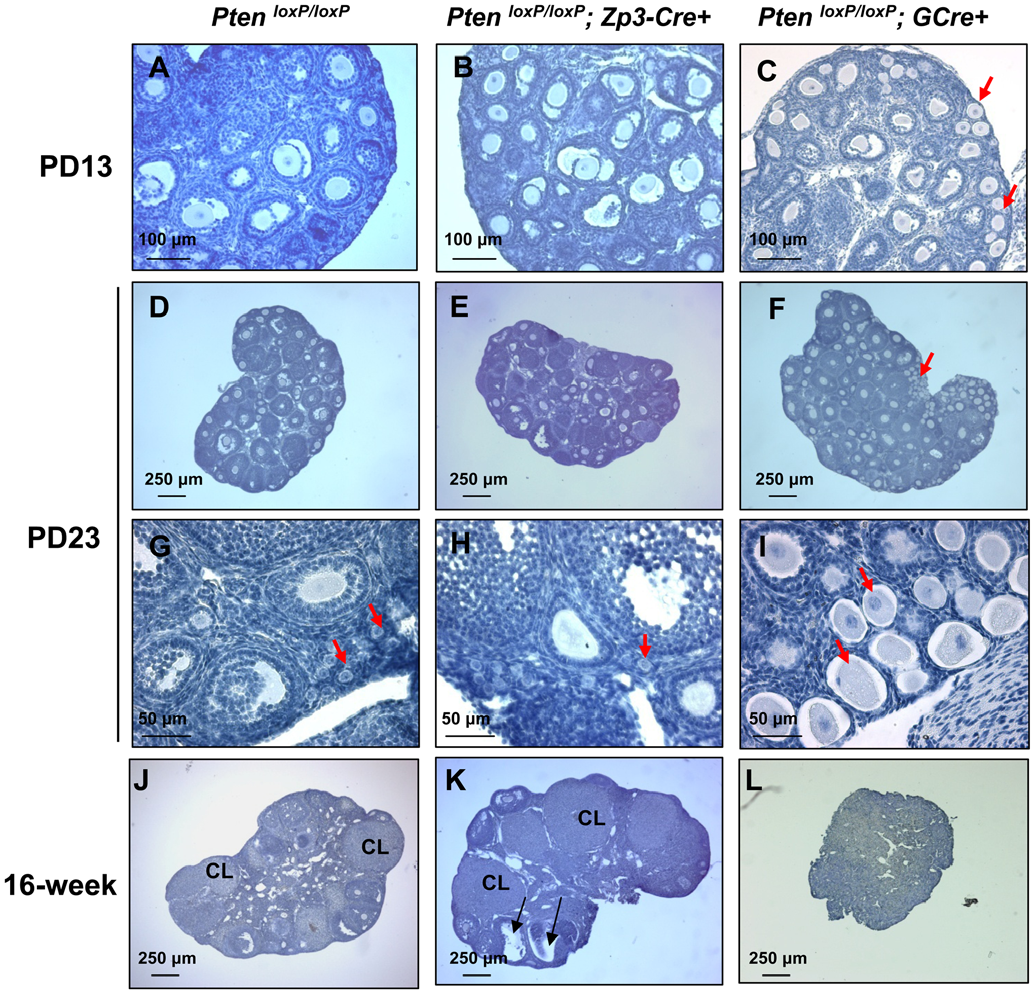
Normal follicular development in *Pten^loxP/loxP^*; *Zp3-Cre+* mice. Morphological analysis of ovaries from 13- and 23-day-old, and 16-week-old *Pten^loxP/loxP^*; *Zp3-Cre+* mice, *Pten^loxP/loxP^*; *GCre+* mice, and control *Pten^loxP/loxP^* mice. Ovaries were embedded in paraffin and sections of 8-µm thickness were prepared and stained with hematoxylin. Note the overactivation of primordial follicles in *Pten^loxP/loxP^*; *GCre+* ovaries (C, F, and I, arrows) and the normal follicular development and CL in *Pten^loxP/loxP^*; *Zp3-Cre+* ovaries (B, E, H and K), which is comparable to the control *Pten^loxP/loxP^* ovaries (A, D, G, and J). CL, corpora lutea.

Our previous work has shown that deletion of *Pten* from oocytes of primordial follicles leads to activation of the entire pool of primordial follicles, which eventually results in follicle depletion and premature ovarian failure (POF) in young adulthood [6]. To determine the function of *Pten* in oocytes of primary follicles, we first examined ovarian morphology of *Pten^loxP/loxP^*; *Zp3-Cre+* at postnatal day 13 (PD13), PD23, and at 16 weeks of age in comparison to control *Pten^loxP/loxP^* ovaries. We found that *Pten^loxP/loxP^*; *Zp3-Cre+* ovaries at PD13 appeared normal in morphology as compared to control *Pten^loxP/loxP^* ovaries, with primordial, primary, secondary, and some early antral follicles (Fig. 4, A and B). In comparison, in ovaries of mice with deletion of *Pten* in oocytes of primordial follicles (referred to as *Pten^loxP/loxP^*; *GCre+* mice), prematurely activated primordial follicles (transient follicles with enlarged oocytes surrounded by flattened pre-granulosa cells) were observed (Fig. 4C, red arrows), as reported earlier [6]. These results show that deletion of *Pten* from primary follicles does not affect the primordial follicles in *Pten^loxP/loxP^*; *Zp3-Cre+* mice.

By PD23, all the primordial follicles were activated in *Pten^loxP/loxP^*; *GCre+* ovaries (Fig. 4, F and I, arrows). However, *Pten^loxP/loxP^*; *Zp3-Cre+* ovaries exhibited normal morphology with primordial follicles (Fig. 4H, red arrows) and growing follicles (Fig. 4, E and H), which were comparable to *Pten^loxP/loxP^* ovaries (Fig. 4D; Fig. 4G, arrows). The premature activation of primordial follicles in *Pten^loxP/loxP^*; *GCre+* mice led to complete depletion of follicles by 16 weeks of age (Fig. 4L) [6]. However, in ovaries of *Pten^loxP/loxP^*; *Zp3-Cre+* mice at 16 weeks of age, the morphology was normal with all types of follicles and corpora lutea (CL) (Fig. 4K), which was similar to *Pten^loxP/loxP^* ovaries (Fig. 4J). Specifically, healthy CL and preovulatory follicles (Fig. 4K, arrows) were observed in 16-week-old *Pten^loxP/loxP^*; *Zp3-Cre* ovaries.

### Normal resumption of meiosis in *Pten^loxP/loxP^*; *Zp3-Cre+* oocytes

**Figure 5:**
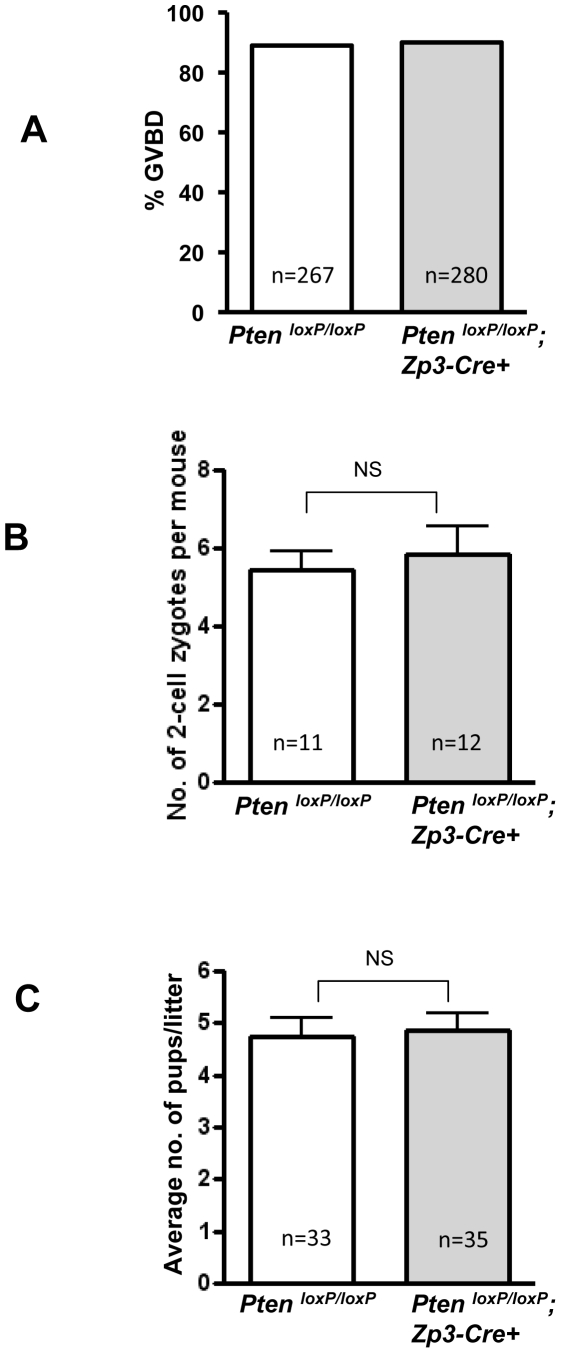
Normal GVBD, ovulation, fertilization, and fertility in *Pten^loxP/loxP^*; *Zp3-Cre+* mice. (A) Oocytes from 3–4-week-old PMSG-primed *Pten^loxP/loxP^*; *Zp3-Cre+* mice or control *Pten^loxP/loxP^* mice were collected in M2 medium. They were cultured further in M16 medium for GVBD analysis. GVBD rate was scored after a culture period of 6 h. Normal GVBD rates were observed in *Pten* mutant oocytes as compared to control oocytes. The numbers of oocytes used (n) are shown. (B) Two-cell embryos were collected at E1.5 and were cultured in KSOM medium supplemented with amino acids. Numbers of 2-cell embryos per female (mean±SEM) mouse were counted. The numbers of mice used (n) and the results of statistical analysis are shown. No significant difference (NS) was seen between *Pten^loxP/loxP^*; *Zp3-Cre+* mice and control mice. (C) Normal litter size of *Pten^loxP/loxP^*; *Zp3-Cre+* females as compared to *Pten^loxP/loxP^* control mice (mean±SEM). The numbers of litters (n) from 10 mice of each genotype and results of statistical analysis are shown. NS, not statistically significant.

It has been suggested that resumption of meiosis in oocytes is regulated by the PI3K–Akt signaling pathway [9]–[11]. Resumption of meiosis in oocytes involves germinal vesicle breakdown (GVBD), chromatin condensation and segregation, and extrusion of the first polar body [12]. To determine whether the enhanced PI3K–Akt signaling has any affect on resumption of meiosis in oocytes, we examined the rates of GVBD in *Pten^loxP/loxP^*; *Zp3-Cre+* oocytes. Surprisingly, we found that the rate of GVBD in *Pten*-deficient oocytes was unaltered as compared to the control oocytes (Fig. 5A). These data indicate that the resumption of meiosis in oocytes was not affected by elevated PI3K–Akt signaling in *Pten^loxP/loxP^*; *Zp3-Cre+* oocytes.

### Normal ovulation, fertilization, and fertility in *Pten^loxP/loxP^*; *Zp3-Cre+* mice

To test whether overactivation of PI3K–Akt signaling in oocytes has any effect on ovulation, *Pten^loxP/loxP^*; *Zp3-Cre+* female mice and control *Pten^loxP/loxP^* female mice were mated with wild-type males. Two-cell stage zygotes were retrieved from oviducts at E1.5 and were counted. We found that the numbers of 2-cell embryos in *Pten^loxP/loxP^*; *Zp3-Cre+* mice were similar to those in control *Pten^loxP/loxP^* mice (Fig. 5B), indicating that the rates of ovulation and fertilization are not affected by the loss of *Pten* in ovulated oocytes. Importantly, during a testing period from 6 to 30 weeks of age, the *Pten^loxP/loxP^*; *Zp3-Cre+* mice were completely fertile, with normal litter sizes (Fig. 5C).

The above results show that the elevated PI3K–Akt signaling in oocytes caused by loss of *Pten* does not affect follicular development, ovulation, and fertility in *Pten^loxP/loxP^*; *Zp3-Cre+* mice.

## Discussion

In recent years, the PI3K pathway in primary oocytes has been suggested to have important roles in controlling the activation and early development of ovarian follicles [4], [13]–[15]. Activation of the PI3K pathway in oocytes by somatic cell-produced growth factors, such as Kit ligand (KL, or stem cell factor, SCF), results in activation of Akt, which is thought to mediate oocyte growth during follicular activation and early development [15]. Our previous study provided direct functional evidence that the initial recruitment of primordial follicles is tightly regulated by PI3K signaling in oocytes, as oocyte-specific deletion of *Pten* at the primordial follicle stage leads to global activation of all primordial follicles [6].

In the present study, to investigate how the PTEN/PI3K–Akt signaling in oocytes affects follicular development and oocyte maturation we deleted *Pten* in oocytes of primary and further developed follicles by using *Zp3* promoter-mediated Cre transgenic mice. As expected, we found that the pool of primordial follicles was unaffected throughout the reproductive life of *Pten^loxP/loxP^*; *Zp3-Cre+* mice. Moreover, we observed normal follicular development, ovulation, and fertility in *Pten^loxP/loxP^*; *Zp3-Cre+* mice, indicating that the PI3K signaling in oocytes is essential for follicular activation in a stage-specific fashion.

Foxo3a, which is a transcription factor and also an Akt substrate, has been shown to suppress follicular activation [16]. Oocyte-specific deletion of *Foxo3a* and *Foxo3a*-deficient (*Foxo3a^−/−^*) mice show similar phenotypes of premature activation of the entire primordial follicle pool [16], [17], as we demonstrated in *Pten^loxP/loxP^*; *GCre+* mice [6]. Interestingly, Foxo3a was found to be specifically required for the prevention of primordial follicles from activation [18]. Thus, these results support the notion that the PI3K signaling in oocytes is probably more important for follicular activation in a stage-specific way.

It has been suggested that PI3K–Akt signaling plays an important role in the meiotic maturation of oocytes [9]–[11], [19]. Inhibition of Akt activation in oocytes by a PI3K-specific inhibitor LY294002 results in suppressed GVBD and polar body extrusion in mouse oocytes [19], and inhibition of Akt phosphorylation leads to inactivation of cyclin-dependent kinase 1 (Cdk1), which in turn causes inhibition of meiosis resumption [11]. Furthermore, activation of phosphodiesterase 3A (PDE3A) by Akt-mediated phosphorylation has been suggested to play a role in the control of PDE3A activity in the maturation of mammalian oocytes [9]. The above studies suggest the importance of PI3K–Akt signaling in the oocyte for resumption of meiosis and maturation of mouse oocytes. At the same time, however, results from the current study have shown that oocytes from *Pten^loxP/loxP^*; *Zp3-Cre+* mice undergo normal maturation, ovulation, and fertilization, and the mutant mice were completely fertile with normal litter sizes, indicating that the loss of *Pten* in oocytes does not apparently affect the fertility of the mice.

Results from our current work with the two mutant mouse models, the *Pten^loxP/loxP^*; *GCre+* mice and *Pten^loxP/loxP^*; *Zp3-Cre+* mice that carry *Pten* deficiency in oocytes of primordial and primary follicles, respectively, indicate that PTEN/PI3K–Akt signaling in oocytes is critically important for maintenance of the primordial follicle pool. On the other hand, further detailed and preferably *in vivo* studies are needed to elucidate the roles of PTEN and PI3K signaling in oocytes during oocyte maturation.

## Materials and Methods

### Mice

The *Pten^loxP/loxP^* mice [8] and *Zp3-Cre* transgenic mice where the Cre recombinase is specifically expressed in oocytes of primary and further developed follicles (Fig. 1) [7], were obtained from the Jackson Laboratory (Bar Harbor, MN). Both strains of mice have a C57BL/6J genomic background. After multiple rounds of crossing, mice with *Pten^loxP/loxP^*; *Zp3-Cre+* genotype and control female mice with a *Pten^loxP/loxP^* genotype were obtained for experiments.

The mice with deletion of *Pten* in oocytes of primordial follicles (referred to as *Pten^loxP/loxP^*; *GCre+* mice) were obtained by crossing *Pten^loxP/loxP^* mice with transgenic mice carrying *Gdf-9* promoter-mediated Cre recombinase, which is specifically expressed in oocytes of primordial and further developed follicles (Fig. 1) [20], as previously described [6].

The mice were housed under controlled environmental conditions with free access to water and food. Illumination was on between 0600 and 1800 h. All experiments were conducted with the approval of the regional ethical committee of Umeå University, Sweden.

### Reagents, antibodies, and immunological detection methods

The rabbit polyclonal antibodies to Akt, phospho-Akt (Ser473), tuberin/Tsc2, rabbit monoclonal antibody to phospho-tuberin/Tsc2 (Thr1462), and mouse monoclonal antibody to PTEN were obtained from Cell Signaling Technologies (Beverly, MA). Mouse monoclonal antibody to phospho-Akt (Thr308) was purchased from BD Bioscience (Franklin Lakes, NJ). PMSG and mouse monoclonal antibody to β-actin were purchased from Sigma-Aldrich Sweden AB (Stockholm, Sweden). Western blots were carried out according to the instructions of the suppliers of the different antibodies and were visualized using the ECL Plus Western Blotting Detection System (Amersham Biosciences, Uppsala, Sweden).

### Histological analysis

Ovaries were fixed in 4% paraformaldehyde, dehydrated, and embedded in paraffin. The paraffin-embedded ovaries were sectioned at 8-µm thickness and stained with hematoxylin for morphological observation. Ovarian follicles at different developmental stages were categorized based on the well-accepted standards established by Pedersen and Peters [21]. Growing transient follicles were defined as follicles that have obviously enlarged oocytes but which are still enclosed in flattened pre-granulosa cells. Morphology images were taken with a light microscope (Leica).

### Oocyte collection and culture

To stimulate follicular development, immature 23-day-old female mice were injected intraperitoneally with 5 IU of PMSG. The ovaries were collected 48 h later and were dissected free of fat and connective tissue. Oocytes were collected by puncturing the ovaries with a sharp needle in M2 medium (Sigma), selected using a mouth pipette, and then cultured further in M16 medium (Sigma) at 37°C in an atmosphere of 5% CO_2_ for 6 h to determine the rate of GVBD.

For western blot, oocytes were lysed immediately after being selected with a mouth pipette, in a buffer containing 50 mM Tris-HCl (pH 8.0), 120 mM NaCl, 20 mM NaF, 20 mM β-glycerophosphate, 1 mM EDTA, 6 mM EGTA (pH 8.0), 1% NP-40, 1 mM DTT, 5 mM benzamidine, 1 mM PMSF, 250 µM sodium vandadate, 2 µg/ml aprotinin, 10 µg/ml leupeptin, and 1 µg/ml pepstatin, for 20 min on ice with frequent vortexing. The oocyte extracts were then used for western blot.

### Natural ovulation and embryo culture

Adult female mice were housed with wild-type males, and vaginal plugs were checked every morning. Embryonic day 0.5 (E0.5) refers to the day when a vaginal plug was found. The mated females were sacrificed at E1.5, and 2-cell embryos were recovered from their oviducts and cultured further in KSOM medium (Specialty Media). The numbers and stages of development of the embryos were recorded.

### Statistical analysis

All experiments were repeated at least 3 times. For comparisons of oocyte numbers, differences between the two groups were calculated with Student's t-test, and a difference was considered significant if *P*<0.05.
